# Role of H-FABP values in determining the etiologic factors of the cardiac injuries

**DOI:** 10.11604/pamj.2017.26.36.8746

**Published:** 2017-01-24

**Authors:** Guleser Akpinar, Ali Duman, Bedia Gulen, Mucahit Kapci, Ertugrul Altinbilek, Ibrahim Ikizceli

**Affiliations:** 1 Sisli Hamidiye Etfal Training and Research Hospital, Department of Emergency Medicine, Istanbul, Turkey; 2 Adnan Menderes University Hospital, Department of Emergency Medicine, Aydin, Turkey; 3 Bezmialem Vakif University Medical School, Department of Emergency Medicine, Istanbul, Turkey

**Keywords:** H-FABP, cardiac injury, thoracic trauma

## Abstract

**Introduction:**

Cardiac injury resulting from blunt thoracic trauma is a frequent clinical occurrence which is difficult to diagnose. Our purpose in this study was to research whether H-FABP, which is a new marker for the diagnosis of cardiac injury, can be used in this patient group.

**Methods:**

50 patients with blunt thoracic injury who were admitted to our emergency service within a period of 8 months and 50 cases as controls were included in our study.

**Results:**

Of the 50 patients with blunt thoracic injury in our study, 88% were male while 12% were female. The average age of the patients was 43 ± 15.15. While 27 (54%) of the 50 patients with blunt thoracic injury had cardiac injury, 23 (46%) did not have cardiac injury. The results of the statistical analyses showed a significant association between thorax trauma and cTnI, CPK, CPKMB and H-FABP (p<0.05). While there was a significant association between cardiac injury resulting from thoracic trauma and cTnI, ECG and TTE (p<0.05), there was no significant association between CPK, CPKMB and H-FABP (p>0.05).

**Conclusion:**

In thoracic traumas, cardiac injury diagnosis can be made as a result of the assessment with Troponin-I, ECG and ECHO. For cardiac injury diagnosis, wide scale prospective studies are needed for H-FABP use.

## Introduction

Today, traumas are one of the most important public health issues. About 1/3 of the patients hospitalized for traumas are severe thoracic traumas. Although the heart is thought to be well protected inside the thoracic cage, blunt cardiac injury occurs in 20 to 70% of motor vehicle accidents. Cardiac injury resulting from blunt thoracic trauma is a frequent clinical occurrence which is difficult to diagnose. Cardiac injuries can occur in a wide spectrum ranging from asymptomatic myocardial injury to rupture and death. Generally, there may not be a correlation between the severity of the trauma and cardiac injury. There is no single golden standard test for diagnosis of cardiac injury [1]. Since heart-type fatty acid binding protein (H-FABP) diffuses to plasma rapidly following myocardial injury, it is a biochemical marker which has come to the forefront recently in the early diagnosis of cardiac injury [2]. Its concentration increases in the first 1.5 hours, reaches the peak point within 5-6 hours and tends to decrease after 6 hours. It returns to normal levels 24-30 hours later. It is thought to have an important place in the diagnosis of myocardial injury [3]. Apart from myocardium, it is found in skeleton muscle, brain, mammary gland and placenta. There are recent studies which state that it can be used in the early diagnosis of especially acute coronary syndrome, coronary failure, kidney and liver injury, pulmonary embolism and some poisonings [4]. In this study, our purpose was to asses H-FABP, a new marker for the cardiac injury diagnosis in patients with thoracic trauma by using cardiac troponin (cTnI), creatine phosphokinase (CPK), creatine phosphokinase myocardial band (CPKMB), transthoracic echocardiography (TTE) and electrocardiography (ECG).

## Methods

After obtaining the approval of the Ethics Committee (2008/342), we have evaluated 50 cases of blunt chest traumas reported in the Erciyes University Medicine School Academic Emergency Department during the dates between 01.11.2008 and 15.07.2009. Of the 50 patients with thoracic injury in our study, cardiac injury was diagnosed in 27 patients. Department of our While blood was drawn in each trauma case for biochemical parameters, 12 lead ECGs of the patients were also recorded. Necessary radiographies and PA chest X-rays, in particular, have been recorded with respect to the vital signs and cases. The biochemical parameters that were being worked on at the time of the patient’s admission were CPK, CKMB, cTnI, H-FABP, ECG respectively. Those values were worked on at the time of their arrival in the emergency services and 6th, 12th and 24th hours thereafter. The cases that cause an increase in the CKMB, cTnI, HFABP levels (such as the patients with polymyositis, dematomyositis, chronic renal failure, and those who have undergone intramuscular injection in the last 24 hours and those with cardiopulmonary resuscitation history) were left out of the scope of the study. Admission criteria for the study are as follows: rib fracture; sternum or vertebrae fracture; presence of hemothorax, pneumothorax or hemopneumothorax; patients with pulmonary damage in their PA chest X-ray.

Trauma Severity Score was used when the severity of the trauma was estimated. All the biochemical parameters were studied through venous blood serums that were taken 1 hour after the occurrence of the trauma case at the earliest, and 3-4 hours on average and 8 hours at the latest. Echocardiography scans of the cases were taken during the first 24 hours by Erciyes University Medicine School Cardiology Department. Cardiology and emergency department doctors by thoracic trauma patients from 50 clinical examination, ECG, TTE findings and assessed with cTnI after cardiac injury was diagnosed in 27 patients. 50 healthy patients participating in the study on a voluntary basis who had no blunt thorax trauma were considered to be the control group. Blood samples were taken from each member of the control group for determining their H-FABP.

The “SPSS 15.0 for Windows” package program was used for the statistics used in the study. Chi-squre test was conducted for the comparison of the categorical data whereby those whose p value equaling 0.05 or less were considered to be positive values. Mann-Whitney test was employed in the correlation evaluation.

## Results

Of the 50 patients with thoracic injury in our study, 44 (88%) were male while 6 (12%) were female. The average age of the patients with thoracic injury was 43 ± 15.15. 24(48%) of the patients in the control group were male, while 26 (52%) were female. The average age of the controls was 25± 9.53. Of the 50 patients with thoracic trauma, 26 (52%) were admitted to the emergency service due to in-vehicle traffic accident, 14 (28%) were admitted due to extravehicular traffic accident, 7 (14%) were admitted due to falling and 3 (6%) were admitted due to assault. While 45 of the 50 patients with thoracic trauma were hospitalized, 5 were discharged from the emergency service. 1 of the hospitalized patients died on the second day following the trauma.

According to the results of the radiographic assessments of 50 patients with thoracic trauma; 30 patients had rib fracture, 7 patients had vertebral fracture, 4 patients had sternum fracture, 25 patients had hemothorax, 4 patients had scapular fracture, 14 patients had pneumothorax, 13 patients had contusion in the lungs. While 27 (54%) of the 50 patients with blunt thoracic injury had cardiac injury, 23 (46%) did not have cardiac injury. Injury severity score (ISS) averages of the thoracic trauma patients who had cardiac injury was 19 (14-30), while ISS averages of the thoracic trauma patients who did not have cardiac injury was 17 (10-21). According to statistical analysis, there was no significant association between cardiac injury resulting from thoracic trauma and ISS (p>0.05) (Table 1).

**Table 1:** Patient’s ECG, TTE and laboratory findings according to the cardiac injury

	Cardiac injury (-)	Cardiac injury (+)	p
ECG	12 (%24)	32 (%76)	0,003
TTE	11 (%22)	39 (%78)	0,001
H-FABP	61,25 (21,00-112,00)	59,00 (18,00-95,00)	0,471
cTnI	0,03 (0,02-0,07)	0,01 (0,01-0,02)	0,000
CPK	872,00(391,00-13,00)	491,00 (283,00-1419,00)	0,676
CPKMB	55,00 (37,00-90,00)	73,00 (30,00-98,00)	0,930
ISS	19,00 (14,00-30,00)	17,00 (10,00-21,00)	0,073

H-FABP: heart-type fatty acid binding protein

cTnI: cardiac troponin

CPK: creatine phosphokinase

CPKMB: creatine phosphokinase myocardial band

TTE: transthoracic echocardiography

ECG: electrocardiography

ISS: injury severity score

When all the patients were assessed in terms of ECG results, 12 (24%) patients were found to have ECG change, while 38 (76%) patients were not found to have ECG change. In 11(40,7%) of the patients with cardiac injury, significant change was found in ECG. Statistical analysis showed significant association between cardiac injury resulting from thoracic trauma and ECG (p<0.05). When all the patients were assessed in terms of TTE results, 11 (22%) patients had pathology in TTE, while 39 (78%) patients did not have pathologic change in TTE. 11 (40,7%) of the 27 patients with cardiac injury were found to have significant results in TTE. A significant association was found between cardiac injury resulting from thoracic trauma and TTE (p<0.05) (Table 1). cTnI, CPK, CPKMB and H-FABP values of the patients with thoracic trauma and the controls were compared. Statistical results showed significant association between thoracic trauma and cTnI, CPK, CPKMB and H-FABP (p<0.05) (Table 2).

**Table 2:** Patient’s ECG, TTE and laboratory findings according to the cardiac injury

	Blunt thoracic injury	Control group	p
HsFABP	60,12(1,17-2,42)	1,60 (18,00-106,00)	0,000
Troponin	0,02 (0,01-0,04)	0,01 (0,01-0,01)	0,000
CPK	608,00 (347,50-1447,25)	105,00 (74,50-129,25)	0,000
CKMB	55,50 (31,00-92,00)	11,50 (10,0-14,00)	0,000

H-FABP: heart-type fatty acid binding protein

cTnI: cardiac troponin

CPK: creatine phosphokinase

CPKMB: creatine phosphokinase myocardial band

Of the 50 patients with thoracic injury in our study, cardiac injury was diagnosed in 27 patients. In patients with thoracic injury, cardiac injury was assessed in terms of cTnI, CPK, CPKMB and H-FABP. Statistical results showed a significant association between cardiac injury resulting from thoracic trauma and cTnI (p<0.05), while no significant association was found in terms of CPK, CPKMB and H-FABP (p>0.05) (Table 1).

## Discussion

One of the most important and also most neglected injuries following a trauma is cardiac injury resulting from thoracic injury. Although the mechanism of cardiac injury in blunt thoracic trauma is not completely understood, it is thought that it occurs as a result of heart being stuck between the vertebral colon and sternum or as a result of increased thoracic pressure being transmitted to cardiac cavities [5].

In a study by Çobanoğlu et al., most of the patients (79,5%) were men and the average age of all the patients was 38 ± 35 [6]. In Emet et al.’s study, average age of the patients was 47.6 ± 21.5 and most of the patients (81.8%) were men [7]. In our study, most of the patients (88%) were men and the average age of all the patients was 43 ± 15.15, which was in parallel with the literature. These results show that in these decades, people take part in active life more, and just like the general society, men in our country have a more active life in social life.

Taşdemir et al. stated the most common reasons of blunt thoracic trauma as motor vehicle accidents and falling from high [8]. In Skinner et al.’s study, the most common reason was motor vehicle accidents with 92% [9]. Emet et al. also stated the most common reason as motor vehicle accidents (71%) and falling from high (23%) [7]. In line with the literature, the most common reason in our study was motor vehicle accidents.

In their study, Skinner et al. found that ISS averages in patients who did not have cardiac injury was 31 (21-38), while ISS averages of the thoracic trauma patients who had cardiac injury was 37 (29-47) and the result was statistically significant [9]. ISS average in our study was lower and it was not statistically significant. This difference can be explained with the less number of multiple traumas in our patients.

When the thoracic trauma diagnoses in Emet et al.’s study were examined, the following results were found: rib fractures (83%), pneumothorax (39%), hemothorax (31%), lung contusion (13%), clavicular fracture (13%), hemopneumothorax (19%), sternum fracture (10%), scapula fracture (6%), flail chest (3%), and thoracic vertebra fracture (2%) [7]. The results of Skinner et al.’s study were as follows: Pulmonary contusion (%82), Rib fractures (%43), Sternal fracture (%5), Blunt aortic injury (%5), Flail segment (%24) [9]. The results of our study were similar to the results in literature. The incidence of cardiac injury resulting from blunt chest trauma in literature varies between 8% and 76% [10]. The incidence of cardiac injury was 50% in Skinner et al.’s study [9]. 54% cardiac injury was found in our study, which was in parallel with the literature.

The rate of positive finding in echo has been reported as 13–38% in literature [11]. In their study, Emet et al. performed TTE on 34% of the patients and found cardiac injury in 27.3% of these patients. Skinner et al. performed TTE on 45% of the patients and found abnormal results in 79% of these patients [9]. In our study, TTEs of 11 (22%) patients who had thoracic trauma had pathological findings, while TTEs of 11 (40.7%) patients who had cardiac injury showed significant results. Differences between studies can be explained with TTE not being performed on all patients due to limitations.

In their study, Emet et al. found ECG change in 41.4% of the patients who had cardiac injury [7]. In their retrospective study, Skinner et al. found ECG change in 17% of the patients [9]. It was stated that the rates were low since their study was retrospective. In our study, ECG change was found in 40.7% of the patients with cardiac injury. Significant association was found between cardiac injury and ECG in our statistical analyses (p<0.05). It is difficult to find out cardiac injury with only one ECG. The fact that there is no ECG change although there is cardiac injury is an important finding. In trauma patients, ECG may show nonspecific changes due to hypoxia, hypovolemia, anemia, failures in serum electrolytes, vagal or sympathic tonus. Previous ECG changes of the patients and ECG changes which are normal for the patients but accepted as positive findings increase insecurity to this parameter [12, 13].

As we included increased Tn-I level within the diagnostic criteria for BCI, the specificity for Tn-I was 100%; however, its sensitivity was 68.2%. The diagnostic value of abnormal CPK-MB/CPK ratio in determining BCI was low [7]. Bertinchant et al. followed 94 patients with blunt chest trauma and found that troponin I had a sensitivity of only 23%, with a specificity of 97% [14]. In our study, there was a significant association between cardiac injury resulting from thoracic trauma and cTnI, which was in parallel with the literature (p<0.05).

According to literature, since CPK increases as a result of trauma and liver, diaphragm and intestinal injuries can also increase with serious skeleton muscle injuries, they are not specific. While Emet et al. found the sensitivity of CPK and CPKMB (90,9%-95,4%) high in their study, they found that their specificity (4,6%-6,1%) was not high enough for diagnosis [7]. In our study, while CPK and CPKMB were statistically significantly high in thoracic traumas, they were not significantly high in cardiac injury, which was in line with the literature.

H-FABP is relatively small nuclear weighted cytoplasmic protein. It constitutes 2-5% of the cytosolic protein of the heart muscle. A great number of studies have shown it to be a sensitive early marker for myocardial injury in acute coronary syndrome [3]. While there are studies in literature about coronary syndrome, heart failure, pulmonary emboli and pulmonary hypertension in literature, there aren’t enough studies about cardiac injury resulting from trauma. In our study, while there was a statistically significant association between H-FABP and thoracic trauma, no significant association was found between H-FABP and cardiac injury.

## Conclusion

In the diagnosis of cardiac injury in thoracic traumas, cTn-I, ECG and TTE are not enough to make a diagnosis or to rule out a diagnosis. As it has been suggested in literature, for the diagnosis of cardiac injury, first of all there should be a suspicion and clinical examination findings should be assessed with cTn-I, ECG and TTE. In our study, H-FABP was found to be high in 41 of the 50 patients. However, when the results were statistically assessed, they were not significantly high in patients with cardiac injury. Multi centered prospective studies with 24-hour monitoring are needed for H-FABP use in the diagnosis of cardiac injury.

### What is known about this topic

- Cardiac injury resulting from blunt thoracic trauma is a frequent clinical occurrence which is difficult to diagnose. There is no single golden standard test for diagnosis of cardiac injury still. H-FABP diffuses to plasma rapidly following myocardial injury.

### What this study adds

- While there was a statistically significant association between H-FABP and thoracic trauma, no significant association was found between H-FABP and cardiac injury.
